# Nonlocal Strain Gradient Theory for the Bending of Functionally Graded Porous Nanoplates

**DOI:** 10.3390/ma15238601

**Published:** 2022-12-02

**Authors:** Rabab A. Alghanmi

**Affiliations:** Department of Mathematics, College of Sciences and Arts, King Abdulaziz University, Rabigh 21911, Saudi Arabia; raalghanmi@kau.edu.sa

**Keywords:** porosity, nonlocal strain gradient theory, bending, functionally graded material, Navier method, industrial development

## Abstract

Many investigators have become interested in nanostructures due to their outstanding mechanical, chemical, and electrical properties. Two-dimensional nanoplates with higher mechanical properties compared with traditional structural applications are a common structure of nanosystems. Nanoplates have a wide range of uses in various sectors due to their unique properties. This paper focused on the static analysis of functionally graded (FG) nanoplates with porosities. The nonlocal strain gradient theory is combined with four-variable shear deformation theory to model the nanoplate. The proposed model captures both nonlocal and strain gradient impacts on FG nanoplate structures by incorporating the nonlocal and strain gradient factors into the FG plate’s elastic constants. Two different templates of porosity distributions are taken into account. The FG porous nanoplate solutions are compared with previously published ones. The impact of nonlocal and strain gradient parameters, side-to-thickness ratio, aspect ratio, and porosity parameter, are analyzed in detail numerically. This paper presents benchmark solutions for the bending analysis of FG porous nanoplates. Moreover, the current combination of the nonlocal strain gradient theory and the four-variable shear deformation theory can be adapted for various nanostructured materials such as anisotropic, laminated composites, FG carbon nanotube reinforced composites, and so on.

## 1. Introduction

Nanostructures have numerous applications for their advantages such as high stiffness and strength compared with weight ratio, elastic modulus greater than 1TPa, and excellent mechanical, chemical, and electrical properties, among others [1]. As a result, they are used in a variety of engineering fields, such as nanoelectromechanical systems, and their low mass and great sensitivity make them ideal for applications in medicine, biosensors, computers, industrial development, and other fields. Several studies have reported on nanostructure mechanical properties [2,3,4,5,6,7,8,9,10,11,12,13,14,15,16,17].

The local (classical) elasticity theory cannot predict the minuscule size influence on nanostructures because of the lack of a nonlocal elasticity theory. Unfortunately, because the material length scale lacks a nonlocal parameter, the local (classical) elasticity theory cannot anticipate the small size influence on nanostructures. Experiments and molecular dynamics (MDs) simulations can be costly, time-consuming, and difficult to model nanostructure systems. Several nonlocal elasticity theories, including Eringen’s nonlocal theory, couple stress theory, strain gradient theory, surface stress theory, and the modified couple stress theory [18,19,20,21,22,23,24,25] have been used to build such atypical plate models to overcome this issue. Using Eringen’s nonlocal relations, Pradhan and Phadikar [26] utilized the classic and first-order shear deformation theories. Via Eringen’s nonlocal linear theory that depends on third-order theory, Aghababaei and Reddy [27] examined the vibration and bending of nanoplates. Shen et al. [28] discussed the vibration of a one-layered graphene sheet via a nanomechanical sensor via the first-order theory. Malekzadeh and Shojaee [29] used a refined plate theory and the differential quadrature technique to analyze the vibration of nanoplates.

Functionally graded materials (FGMs) are a type of composite material of two phases, with the volume percentage of the FGMs elements gradually changing in thickness direction [30]. This property reduces the problems that inhomogeneous composites have with interfaces. FGMs have been enhanced to encompass a wide range of applications. During the manufacturing of FGMs, porosities occur inside the material [31]. Porous FGMs with high stiffness and low density are utilized in a wide range of engineering sectors, including aviation, aerospace, and military applications. Several researchers have studied the FG porous structural mechanical behavior [32,33,34,35,36,37,38,39,40,41,42,43]. Using Eringen’s nonlocal elasticity, Phung-Van et al. [44] demonstrated the influence of porosities on bending and vibration responses for FG nanoplates.

Isogeometric analysis was used by Phung-Van et al. [45] to investigate the nonlinear transient responses of FG porous nanoplates. Dastjerdi and Aghadavoudi [46] discussed the static behavior of sandwich plates with FG nanocomposite face sheets on an elastic basis. The vibration of an isotropic nanoplate under heat load was discussed by Wang et al. [47]. The nonlocal Timoshenko beam theory was used by Simsek and Yurtcu [48] to explain the buckling and bending of FG nanobeams. Aksencer and Aydogdu [49] utilized a Levy type solution for isotropic nanoplate buckling and vibration. Jomehzadeh and Saidi [50] employed the Levy type method to analyze the vibration of an isotropic nanoplate.

Recently, Alghanmi and Zenkour [51] used a four-variable shear and normal deformation theory to investigate the effect of porosity on the static behavior of an FG plate linked to a piezoelectric actuator. Through a modified shear and normal deformation theory, Zenkour and Alghanmi [52] introduced an enhanced porosity distribution for the bending of a new model of FG sandwich plates sitting on Pasternak’s elastic foundation.

According to the above literature reviews, most research concentrates on FG nanoplates and ignores the effect of porosity on the bending response of FG nanoplates. Due to the scarcity of research on the topic of porosities, this study concentrated on demonstrating the impact of the porosity operator. Furthermore, the nonlocal strain gradient integrated was used to investigate the mechanical responses of nanoplates up to this point. However, studies on the bending of FG porous nanoplate by utilizing the nonlocal strain gradient in conjunction with the four-variable shear deformation theory have not been conducted in the literature to date. To the author’s best knowledge, this pairing of the four-variable shear deformation theory and nonlocal strain gradient theory to study FG porous nanoplate is a novel and unexplored topic. The nonlocal parameter and strain gradient parameter, the side-to-thickness ratio, and the porosity and exponent parameters are all discussed.

Validation examples are offered to ensure the current work’s validity. This article is organized as follows: a nonlocal strain gradient model for the bending of FG porous nanoplate is presented in Section 2 with the description of porosities distribution types. Section 3 and Section 4 carry out the governing equations and the solution procedure, respectively. Section 5 provides the numerical results and discussions of the bending analysis of FG porous nanoplate. Section 6 summarizes the study’s main points.

## 2. Basic Formulation

### 2.1. Material Properties of FG Porous Nanoplate

**Figure 1:**
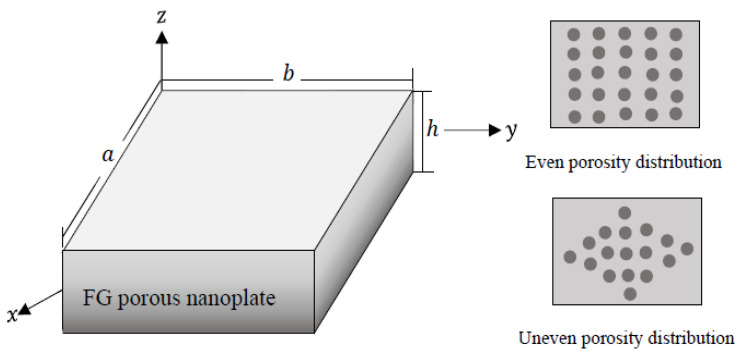
Geometry of FG porous nanoplate.

Consider an FG porous nanoscale plate with thickness h, length a, and width b as depicted in Figure 1. Cartesian coordinates (x,y, z) is considered. The coordinate system is located at the corner of the middle plane of the nanoplate. A distributed mechanical load q(x,y) is acting on the plate top surface (z=h/2). Porosity distributions across the z-axis are presumed to be even and uneven. The material properties of the FG porous nanoplate for porosities with even distribution are described based on the modified power law function as (Daikh and Zenkour [53])

(1) $$P\left(z\right)=P_{m}+\left(P_{c}-P_{m}\right){\left(\frac{z}{h}+\frac{1}{2}\right)}^{k}-\left(P_{c}+P_{m}\right)\frac{\zeta }{2},$$

and according to uneven porosity distribution, the material properties can be written as (Daikh and Zenkour [53])

(2) $$P\left(z\right)=P_{m}+\left(P_{c}-P_{m}\right){\left(\frac{z}{h}+\frac{1}{2}\right)}^{k}-\left(P_{c}+P_{m}\right)\frac{\zeta }{2}\left(1-\frac{2\left|z\right|}{h}\right),$$

where c and m are abbreviations for ceramic and metal, respectively. ζ (0≤ζ≪1) denotes the porosity coefficient and setting α=0 yields the mechanical characteristics for a perfect FG porous nanoplate plate. k denotes the power law exponent (k≥0).

### 2.2. The Nonlocal Strain Gradient Theory for FG Porous Nanoplate

The nonlocal strain gradient theory states that the stress field takes into account both the nonlocal elastic stress field and the strain gradient stress field. The constitutive equation for the FG porous nanoplate can be written as (Askes and Aifantis [54])

(3) $$\left(1-\eta \nabla^{2}\right)\left\{\begin{array}{c} \sigma_{xx}\\ \sigma_{yy}\\ \tau_{yz}\\ \tau_{xz}\\ \tau_{xy} \end{array}\right\}=\left(1-\lambda \nabla^{2}\right)\left[\begin{array}{cc} \begin{array}{c} c_{11}\\ c_{12}\\ \begin{array}{c} 0\\ \begin{array}{c} 0\\ 0 \end{array} \end{array} \end{array} & \begin{array}{ccc} \begin{array}{c} c_{12}\\ c_{22}\\ \begin{array}{c} 0\\ \begin{array}{c} 0\\ 0 \end{array} \end{array} \end{array} & \begin{array}{c} 0\\ 0\\ \begin{array}{c} c_{44}\\ \begin{array}{c} 0\\ 0 \end{array} \end{array} \end{array} & \begin{array}{cc} \begin{array}{c} 0\\ 0\\ \begin{array}{c} 0\\ \begin{array}{c} c_{55}\\ 0 \end{array} \end{array} \end{array} & \begin{array}{c} 0\\ 0\\ \begin{array}{c} 0\\ \begin{array}{c} 0\\ c_{66} \end{array} \end{array} \end{array} \end{array} \end{array} \end{array}\right]\left\{\begin{array}{c} \varepsilon_{xx}\\ \varepsilon_{yy}\\ \gamma_{yz}\\ \gamma_{xz}\\ \gamma_{xy} \end{array}\right\},$$

where $\nabla^{2}=\frac{\partial^{2}}{\partial x^{2}}+\frac{\partial^{2}}{\partial y^{2}}$ is the Laplacian operator. η and λ represent the nonlocal and strain gradient length scale parameters. Setting the strain gradient parameter to zero (λ=0) yields Eringen’s nonlocal model, whereas setting the nonlocal parameter to zero (η=0) yields Kirchhoff’s strain gradient model. The plate stiffness coefficients $c_{ij}$ are written as

(4) $$c_{11}=c_{22}=\frac{E\left(z\right)}{1-\nu^{2}\left(z\right)},\;c_{12}=\frac{\nu \left(z\right)E\left(z\right)}{1-\nu^{2}\left(z\right)},\;c_{44}=c_{55}=c_{66}=\frac{E\left(z\right)}{2\left(1+\nu \left(z\right)\right)},$$

where E(z) is young’s modulus and ν(z) is Poisson’s ratio.

### 2.3. Displacement Model

The displacement model for the FG porous nanoplate is introduced in this study as (Shimpi and Patel [55])

(5) $$\begin{array}{c} u_{1}\left(x,y,z\right)=u\left(x,y\right)-z\frac{\partial w_{b}}{\partial x}-f\left(z\right)\frac{\partial w_{s}}{\partial x},\\ u_{2}\left(x,y,z\right)=v\left(x,y\right)-z\frac{\partial w_{b}}{\partial y}-f\left(z\right)\frac{\partial w_{s}}{\partial y},\\ u_{3}\left(x,y,z\right)=w_{b}\left(x,y\right)+w_{s}\left(x,y\right),\; \end{array}$$

where u and v are the mid-plane displacements. The transverse displacement $u_{3}$ is divided into bending $\left(w_{b}\right)$ and shear $\left(w_{s}\right)$ components. The shape function that specifies the variation in transverse shear stresses across the thickness of the nanoplate is taken as (Thai and Kim [56]); $f\left(z\right)=-\frac{z}{4}+\frac{5}{3}\left(\frac{z^{3}}{h^{2}}\right)$. According to the linear elasticity theory, the strain–displacement relations are provided by

(6a) $$\varepsilon_{ij}=\frac{1}{2}\left(u_{i,j}+u_{j,i}\right)$$

The strain relations based on the mentioned displacement field in Equation (5) can be written as

(6b) $$\begin{array}{c} \varepsilon_{xx}=\frac{\partial u}{\partial x}-z\frac{\partial^{2}w_{b}}{\partial x^{2}}-f\left(z\right)\frac{\partial^{2}w_{s}}{\partial x^{2}},\;\varepsilon_{yy}=\frac{\partial v}{\partial y}-z\frac{\partial^{2}w_{b}}{\partial y^{2}}-f\left(z\right)\frac{\partial^{2}w_{s}}{\partial y^{2}},\\ \gamma_{yz}=\frac{\partial w_{s}}{\partial y}\left[1-f^{\prime }\left(z\right)\right],\;\gamma_{xz}=\frac{\partial w_{s}}{\partial x}\left[1-f^{\prime }\left(z\right)\right],\\ \gamma_{xy}=\frac{\partial u}{\partial y}+\frac{\partial v}{\partial x}-2\left[z\frac{\partial^{2}w_{b}}{\partial x\partial y}+f\left(z\right)\frac{\partial^{2}w_{s}}{\partial x\partial y}\right]. \end{array}$$

## 3. Governing Equations

Using Hamilton’s principle, the following governing equations and associated boundary conditions are derived as follows

(7) $$\;\int_{o}^{a}\int_{0}^{b}\left\{\int_{-\frac{h}{2}}^{\frac{h}{2}}\sigma_{ij}\delta \varepsilon_{ij}dz-{\left[q\left(\delta w_{b}+\delta w_{s}\right)\right]}_{z=-\frac{h}{2}}^{z=\frac{h}{2}}\right\}dydx=0,$$

where q is the distributed transverse load. After replacing the components of strains from Equation (6a) into Equation (7), and thereafter integrating through the z-direction, Equation (7) can be written as

(8) $$\begin{array}{c} \iint_{\Omega }\left\{N_{xx}\frac{\partial \delta u}{\partial x}-M_{xx}\frac{\partial^{2}\delta w_{b}}{\partial x^{2}}-S_{xx}\frac{\partial^{2}\delta w_{s}}{\partial x^{2}}+N_{yy}\frac{\partial \delta v}{\partial y}-M_{yy}\frac{\partial^{2}\delta w_{b}}{\partial y^{2}}-S_{yy}\frac{\partial^{2}\delta w_{s}}{\partial y^{2}}\right.\\ +N_{xy}\frac{\partial \delta u}{\partial y}+N_{xy}\frac{\partial \delta v}{\partial x}-2M_{xy}\frac{\partial^{2}\delta w_{b}}{\partial x\partial y}-2S_{xy}\frac{\partial^{2}\delta w_{s}}{\partial x\partial y}+Q_{yz}\frac{\partial \delta w_{s}}{\partial y}+Q_{xz}\frac{\partial \delta w_{s}}{\partial x}\\ \left.-q\delta w_{b}-q\delta w_{s}\right\}d\Omega =0, \end{array}$$

where the stress resultants $N_{ij}$, $M_{ij},$ $S_{ij},$ and $Q_{iz}$ are defined as

(9) $$\begin{array}{c} \left\{N_{ij},\;M_{ij},S_{ij}\right\}=\int_{-\frac{h}{2}}^{\frac{h}{2}}\sigma_{ij}\left\{1,z,f\left(z\right)\right\}dz,\;i,j=x,y,\\ Q_{iz}=\int_{-\frac{h}{2}}^{\frac{h}{2}}\tau_{iz}\left[1-f^{\prime }\left(z\right)\right]dz,\;i=x,y. \end{array}$$

The governing equations can be derived by the integration of Equation (8) and then collecting the coefficients of $\delta u,\delta v,\delta w_{b},$ and $\delta w_{s}$ as follows

(10) $$\begin{array}{c} \begin{array}{c} \frac{\partial N_{xx}}{\partial x}+\frac{\partial N_{xy}}{\partial y}=0,\\ \frac{\partial N_{xy}}{\partial x}+\frac{\partial N_{yy}}{\partial y}=0, \end{array}\\ \frac{\partial^{2}M_{xx}}{\partial x^{2}}+2\frac{\partial^{2}M_{xy}}{\partial x\partial y}+\frac{\partial^{2}M_{yy}}{\partial y^{2}}+q=0,\\ \frac{\partial^{2}S_{xx}}{\partial x^{2}}+2\frac{\partial^{2}S_{xy}}{\partial x\partial y}+\frac{\partial^{2}S_{yy}}{\partial y^{2}}+\frac{\partial Q_{xz}}{\partial x}+\frac{\partial Q_{yz}}{\partial y}+q=0. \end{array}$$

Inserting the constitutive equations from Equation (3) into Equation (9), the stress resultants for the nanoplate based on the nonlocal strain gradient theory can be expressed as

(11) $$N_{xx}-\eta \nabla^{2}N_{xx}=\left(1-\lambda \nabla^{2}\right)\left(A_{1}\frac{\partial u}{\partial x}-A_{2}\frac{\partial^{2}w_{b}}{\partial x^{2}}-A_{3}\frac{\partial^{2}w_{s}}{\partial x^{2}}+A_{4}\frac{\partial v}{\partial y}-A_{5}\frac{\partial^{2}w_{b}}{\partial y^{2}}-A_{6}\frac{\partial^{2}w_{s}}{\partial y^{2}}\right),\;\;N_{yy}-\eta \nabla^{2}N_{yy}=\left(1-\lambda \nabla^{2}\right)\left(A_{4}\frac{\partial u}{\partial x}-A_{5}\frac{\partial^{2}w_{b}}{\partial x^{2}}-A_{6}\frac{\partial^{2}w_{s}}{\partial x^{2}}+A_{7}\frac{\partial v}{\partial y}-A_{8}\frac{\partial^{2}w_{b}}{\partial y^{2}}-A_{9}\frac{\partial^{2}w_{s}}{\partial y^{2}}\right),\;\;M_{xx}-\eta \nabla^{2}M_{xx}=\left(1-\lambda \nabla^{2}\right)\left(A_{2}\frac{\partial u}{\partial x}-A_{10}\frac{\partial^{2}w_{b}}{\partial x^{2}}-A_{11}\frac{\partial^{2}w_{s}}{\partial x^{2}}+A_{5}\frac{\partial v}{\partial y}-A_{12}\frac{\partial^{2}w_{b}}{\partial y^{2}}-A_{13}\frac{\partial^{2}w_{s}}{\partial y^{2}}\right),\;M_{yy}-\eta \nabla^{2}M_{yy}=\left(1-\lambda \nabla^{2}\right)\left(A_{5}\frac{\partial u}{\partial x}-A_{12}\frac{\partial^{2}w_{b}}{\partial x^{2}}-A_{13}\frac{\partial^{2}w_{s}}{\partial x^{2}}+A_{8}\frac{\partial v}{\partial y}-A_{14}\frac{\partial^{2}w_{b}}{\partial y^{2}}-A_{15}\frac{\partial^{2}w_{s}}{\partial y^{2}}\right),\;S_{xx}-\eta \nabla^{2}S_{xx}=\left(1-\lambda \nabla^{2}\right)\left(A_{3}\frac{\partial u}{\partial x}-A_{11}\frac{\partial^{2}w_{b}}{\partial x^{2}}-A_{16}\frac{\partial^{2}w_{s}}{\partial x^{2}}+A_{6}\frac{\partial v}{\partial y}-A_{13}\frac{\partial^{2}w_{b}}{\partial y^{2}}-A_{17}\frac{\partial^{2}w_{s}}{\partial y^{2}}\right),\;S_{yy}-\eta \nabla^{2}S_{yy}=\left(1-\lambda \nabla^{2}\right)\left(A_{6}\frac{\partial u}{\partial x}-A_{13}\frac{\partial^{2}w_{b}}{\partial x^{2}}-A_{17}\frac{\partial^{2}w_{s}}{\partial x^{2}}+A_{9}\frac{\partial v}{\partial y}-A_{15}\frac{\partial^{2}w_{b}}{\partial y^{2}}-A_{18}\frac{\partial^{2}w_{s}}{\partial y^{2}}\right),\;N_{xy}-\eta \nabla^{2}N_{xy}=\left(1-\lambda \nabla^{2}\right)\left[A_{19}\left(\frac{\partial u}{\partial y}+\frac{\partial v}{\partial x}\right)-2\left(A_{20}\frac{\partial^{2}w_{b}}{\partial x\partial y}+A_{21}\frac{\partial^{2}w_{s}}{\partial x\partial y}\right)\right],\;M_{xy}-\eta \nabla^{2}M_{xy}=\left(1-\lambda \nabla^{2}\right)\left[A_{20}\left(\frac{\partial u}{\partial y}+\frac{\partial v}{\partial x}\right)-2\left(A_{22}\frac{\partial^{2}w_{b}}{\partial x\partial y}+A_{23}\frac{\partial^{2}w_{s}}{\partial x\partial y}\right)\right],\;S_{xy}-\eta \nabla^{2}S_{xy}=\left(1-\lambda \nabla^{2}\right)\left[A_{21}\left(\frac{\partial u}{\partial y}+\frac{\partial v}{\partial x}\right)-2\left(A_{23}\frac{\partial^{2}w_{b}}{\partial x\partial y}+A_{24}\frac{\partial^{2}w_{s}}{\partial x\partial y}\right)\right],\;Q_{yz}-\eta \nabla^{2}Q_{yz}=\left(1-\lambda \nabla^{2}\right)A_{25}\frac{\partial w_{s}}{\partial y},\;Q_{xz}-\eta \nabla^{2}Q_{xz}=\left(1-\lambda \nabla^{2}\right)A_{26}\frac{\partial w_{s}}{\partial x},$$

where the quantities mentioned in the above equations are defined as follows

(12) $$\left[\begin{array}{ccc} A_{1} & A_{2} & A_{3}\\ A_{4} & A_{5} & A_{6}\\ A_{7} & A_{8} & A_{9} \end{array}\right]=\int_{\frac{-h}{2}}^{\frac{h}{2}}\left[\begin{array}{c} c_{11}^{c}\\ c_{12}^{c}\\ c_{22}^{c} \end{array}\right]\left[\begin{array}{ccc} 1 & z & f\left(z\right) \end{array}\right]dz,\;\left[\begin{array}{ccc} A_{10} & A_{11} & A_{16}\\ A_{12} & A_{13} & A_{17}\\ A_{14} & A_{15} & A_{18} \end{array}\right]=\int_{\frac{-h}{2}}^{\frac{h}{2}}\left[\begin{array}{c} c_{11}^{c}\\ c_{12}^{c}\\ c_{22}^{c} \end{array}\right]\left[\begin{array}{ccc} z^{2} & zf\left(z\right) & f^{2}\left(z\right) \end{array}\right]dz,\;\left[\begin{array}{ccc} A_{19} & A_{20} & A_{21}\\ A_{22} & A_{23} & A_{24} \end{array}\right]=\int_{\frac{-h}{2}}^{\frac{h}{2}}c_{66}^{c}\left[\begin{array}{ccc} 1 & z & f\left(z\right)\\ z^{2} & zf\left(z\right) & f^{2}\left(z\right) \end{array}\right]dz,\;\left\{A_{25},A_{26}\right\}=\int_{\frac{-h}{2}}^{\frac{h}{2}}\left\{c_{44}^{c},c_{55}^{c}\right\}{\left[1-f^{\prime }\left(z\right)\right]}^{2}dz.$$

## 4. Closed-Form Solution

The solution is found for a rectangular FG porous nanoplate. The FG nanoplate is assumed to be fully simply supported. The next boundary conditions are imposed on the nanoplate four edges.

(13) $$\begin{array}{c} v=w_{b}=w_{s}=\frac{\partial w_{b}}{\partial y}=\frac{\partial w_{s}}{\partial y}=N_{x}=M_{x}=S_{x}=0,\;\mathrm{at}\;x=0,a,\\ u=w_{b}=w_{s}=\frac{\partial w_{b}}{\partial x}=\frac{\partial w_{s}}{\partial x}=N_{y}=M_{y}=S_{y}=0,\;\mathrm{at}\;y=0,b. \end{array}$$

Following Navier method, assume the solution of the displacement components in the following form (Reddy [57])

(14) $$\left\{\begin{array}{c} \begin{array}{c} u\\ v \end{array}\\ \left(w_{b},w_{s}\right) \end{array}\right\}=\left\{\begin{array}{c} \begin{array}{c} U\cos\left(\alpha x\right)\sin\left(\beta y\right)\\ V\sin\left(\alpha x\right)\cos\left(\beta y\right) \end{array}\\ \left(W_{b},W_{s}\right)\sin\left(\alpha x\right)\sin\left(\beta y\right) \end{array}\right\},$$

where

(15) α=π/a,β=π/b

in which $\left(U,V,\;W_{b},\;W_{s}\right)$ are the unknowns to be determined. A trigonometric development is used for the mechanical load as

(16) $$q=q_{0}\sin\left(\alpha x\right)\sin\left(\beta y\right),$$

where $q_{0}$ denote the concentration of the distributed load at the nanoplate center. 

Substituting Equations (14) and (16) in Equation (10), reveals

(17) [A]{Δ}={F},

where {Δ} and {F} denote the following

(18) $$\begin{array}{c} \left\{\Delta \right\}=\left\{U,\;V,W_{b},W_{s}\right\},\\ \left\{F\right\}=\left\{0,0,q_{0},q_{0}\right\}, \end{array}$$

and the nonzero elements $a_{ij}=a_{ji}$ of the symmetric matrix [A] are in the Appendix A. 

## 5. Results and Discussions

The obtained results are introduced to demonstrate the influence of nonlocal (η) and length scale (λ) parameters on the bending of porous FG plates with even and uneven porosity distributions. The FG porous nanoplate has a length a=10 nm and the constituent materials have the following characteristics

(19) $$E_{m}=70\times 10^{9}\;N/m^{2},\;E_{c}=380\times 10^{9}\;N/m^{2},\;\nu_{m}=\nu_{c}=0.3.$$

### 5.1. Verification Analysis

The accuracy of the present model under the nonlocal effect and without considering strain gradient effect (λ=0) is verified. In this case the nonlocal effect of FG nanoplates is investigated. A validation example compared with Sobhy [58] and Hoa et al. [59] is provided using the following non-dimensional displacement and stresses

(20) $$\begin{array}{c} \bar{w}=\frac{100h^{3}E_{c}}{a^{4}q_{0}}u_{3}\left(\frac{a}{2},\frac{b}{2},\bar{z}\right),\;{\bar{\sigma }}_{1}=\frac{h}{aq_{0}}\sigma_{xx}\left(\frac{a}{2},\frac{b}{2},\bar{z}\right),\\ {\bar{\sigma }}_{5}=\frac{h}{aq_{0}}\sigma_{xz}\left(0,\frac{b}{2},\bar{z}\right),\;{\bar{\sigma }}_{6}=\frac{h}{aq_{0}}\sigma_{xy}\left(0,0,\bar{z}\right),\;\bar{z}=\frac{z}{h}. \end{array}$$

Table 1 shows the non-dimensional deflection and stresses of a local (η=0) and nonlocal (η=2) FG square nanoplate without the effect of porosities for two values of inhomogeneity parameter k. According to this case, the present numerical results are in good agreement to those of Sobhy [59] and Hoa et al. [59], for all cases of nonlocal coefficient and inhomogeneity parameters. Table 2 exhibits the variation in the non-dimensional deflection $\bar{w}$ in a square FG nanoplate (without considering the porosity factor) in terms of the nonlocal coefficient and length-to-thickness ratio. The current findings are consistent with those presented by Hoa et al. [59].

### 5.2. Parametric Analysis

This subsection presents numerical results for the FG porous nanoplate according to the nonlocal strain gradient theory. The influence of different values of the nonlocal parameter (η), length scale parameter (λ), porosity coefficient (ζ), length-to-thickness ratio (a/h), and aspect ratio (a/b) on the bending of the FG porous nanoplate plates are investigated. The dimensionless displacement and stresses used for the present results are

(21) $$\begin{array}{c} \bar{w}=\frac{10h^{3}E_{c}}{a^{4}q_{0}}u_{3}\left(\frac{a}{2},\frac{b}{2},\bar{z}\right),\;{\bar{\sigma }}_{1}=\frac{h}{aq_{0}}\sigma_{xx}\left(\frac{a}{2},\frac{b}{2},\bar{z}\right),\\ {\bar{\sigma }}_{5}=\frac{10h}{aq_{0}}\sigma_{xz}\left(0,\frac{b}{2},\bar{z}\right),\;{\bar{\sigma }}_{6}=\frac{10h}{aq_{0}}\sigma_{xy}\left(0,0,\bar{z}\right),\;\bar{z}=\frac{z}{h}. \end{array}$$

The parameters considered (except otherwise clarified) are k=2,a/h=10, a/b=1,λ=1, η=2. The variation in the non-dimensional deflection and stresses in a square FG porous nanoplate for different values of the nonlocal and length scale parameters are depicted in Table 3, Table 4, Table 5 and Table 6. 

Even and uneven types of porosity distribution with two values of porosity coefficients are discussed in addition to the perfect case. It can be observed from these tables that the center deflection and stresses increase by the decreasing in length scale parameter λ and by the increasing in the nonlocal parameter η, whatever the FG nanoplates type and porosity coefficient ζ are. Furthermore, it can be concluded that as the porosity coefficient ζ increases, so do the deflection $\bar{w}$ and in-plane normal stress ${\bar{\sigma }}_{1}$ as depicted in Table 3 and Table 4. This is due to a decrease in plate stiffness caused by the presence of porosity. Table 5 and Table 6 reveal that the transverse shear stress ${\bar{\sigma }}_{5}$ and the in-plane shear stress ${\bar{\sigma }}_{6}$ are decreased with the existence of porosity factor ζ. It can be concluded that the deflection and stresses results of FG nanoplates with uneven porosities have lower values than the ones with even porosities except for the in-plane stress ${\bar{\sigma }}_{6}$.

**Figure 2:**
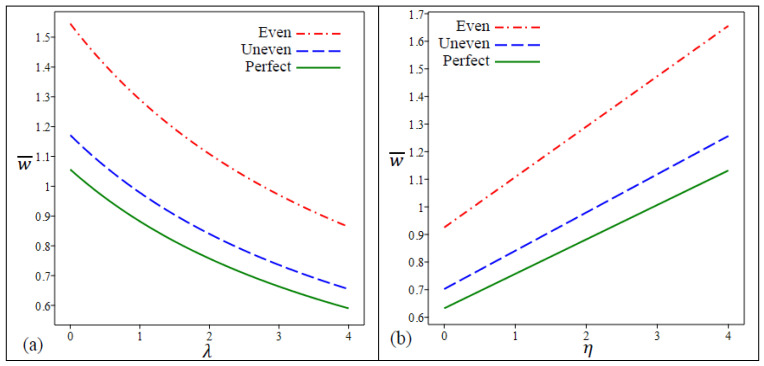
The variation in dimensionless center deflection $\bar{w}$ in a square FG porous nanoplate in terms of (**a**) *λ* (**b**) *η*.

The center deflection $\bar{w}$ variation for three types of FG nanoplates versus the length scale parameter λ and the nonlocal parameter η are displayed in Figure 2a and Figure 2b, respectively. 

**Figure 3:**
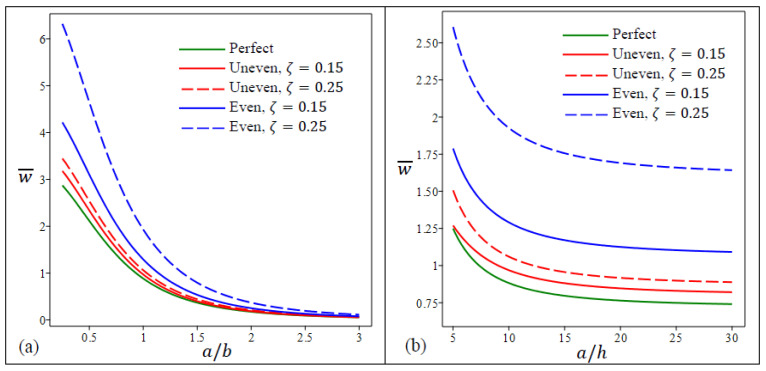
Dimensionless center deflection $\bar{w}$ in a square FG porous nanoplate as a function of (**a**) aspect ratio a/b, (**b**) side-to-thickness ratio a/h.

According to Figure 2, the presence of porosity increases deflection significantly when compared with nonporous FG nanoplates. The nanoplates with porosities that are unevenly distributed deflect less than those that are evenly distributed. Moreover, as previously mentioned, for the three types of FG nanoplates, the center deflection increase by the decreasing in λ and by the increasing in η. In Figure 3a,b, the center deflection $\bar{w}$ variation for five types of FG nanoplates in terms of aspect ratio a/b and length-to-thickness ratio a/h is demonstrated, respectively. The aspect ratio impact on the central deflection is much larger than that of the length-to-thickness ratio for all FG nanoplates types. The FG nanoplates with even porosities (ζ=0.25) have the largest deflections of all types. 

**Figure 4:**
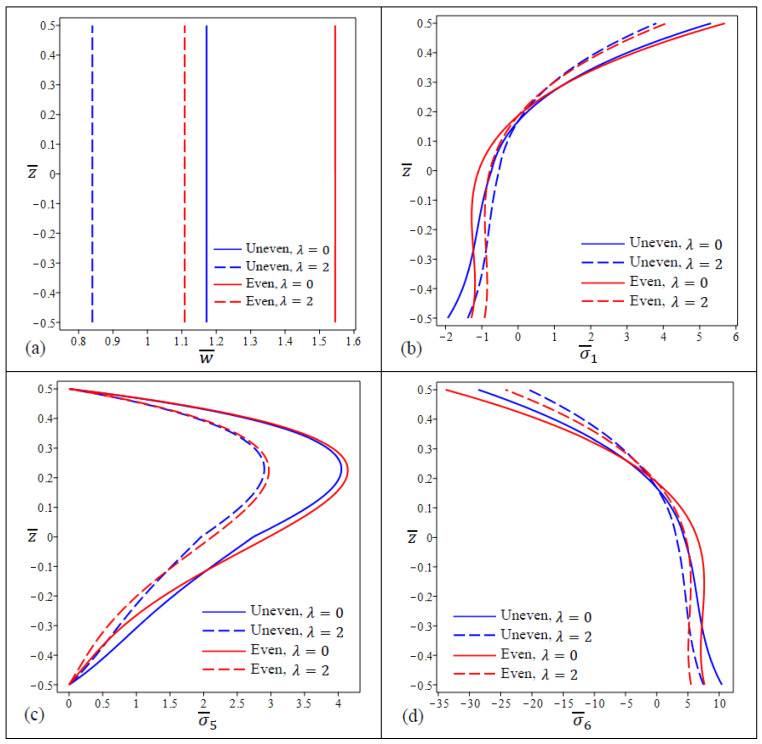
The variation in dimensionless center deflection and stresses in a square FG porous (uneven\even distribution) nanoplate for different values of λ. (**a**) $\bar{w}$, (**b**) ${\bar{\sigma }}_{1}\;$, (**c**) ${\bar{\sigma }}_{5}$, (**d**) ${\bar{\sigma }}_{6}$.

Figure 4 shows the variation in center deflection and stresses for four types of FG porous nanoplate with different values of λ and fixed nonlocal parameter η. The FG nanoplates with uneven porosities and λ=2 have the lowest deflections of all types as shown in Figure 4a. Figure 4b shows that the in-plane normal stress ${\bar{\sigma }}_{1}$ have the lowest values at the FG nanoplate upper surface with the uneven porosity distribution and λ=2 while the even porosity distribution and λ=2 cause the lowest stresses at the lower surface. For FG nanoplates, the variation in shear stress ${\bar{\sigma }}_{5}$ through the thickness is not parabolic, and maximum values do not happen in the center of the plates as shown in Figure 4c.

Furthermore, the FG nanoplates with even and uneven porosities are identical in the upper 15% of the thickness. It can be found from Figure 4d that the in-plane stress ${\bar{\sigma }}_{6}$ for the uneven type are equal to zero at z≅0.18 while for even type are equal to zero at z≅0.19.

**Figure 5:**
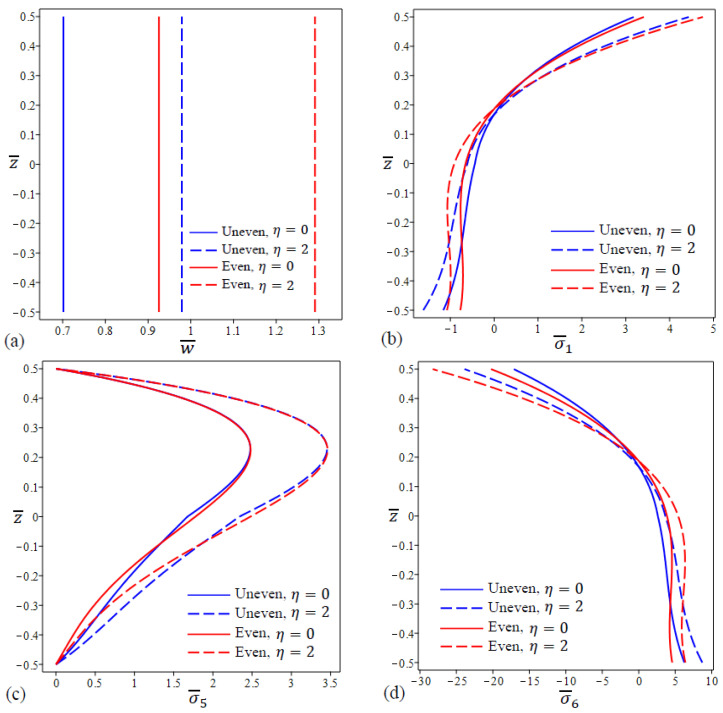
The variation in dimensionless center deflection and stresses in a square FG porous (uneven\even distribution) nanoplate for different values of η. (**a**) $\bar{w}$, (**b**) ${\bar{\sigma }}_{1}\;$, (**c**) ${\bar{\sigma }}_{5}$, (**d**) ${\bar{\sigma }}_{6}$.

With fixed length scale parameter (λ=1) and two different values of the nonlocal parameter (η=0, 2), the variation in center deflection and stresses are depicted in Figure 5 for FG nanoplate with even and uneven porosities. The lowest deflections of all types happened in the case of uneven porosities and η=0 as shown in Figure 5a. The lowest values of in-plane normal stress ${\bar{\sigma }}_{1}$ at the nanoplate upper surface occur with the case of uneven porosity distribution (η=0) while the lowest values of stresses at the nanoplate lower surface happened with the even porosity distribution case (η=0). The maximum values of shear stress ${\bar{\sigma }}_{5}$ occur at z≅0.24 for all types of FG nanoplates as shown in Figure 5c.

It is apparent that the in-plane stresses ${\bar{\sigma }}_{6}$ do not depend on the nonlocal parameter η for both even and uneven cases at z≅0.19 as illustrated in Figure 5d.

**Figure 6:**
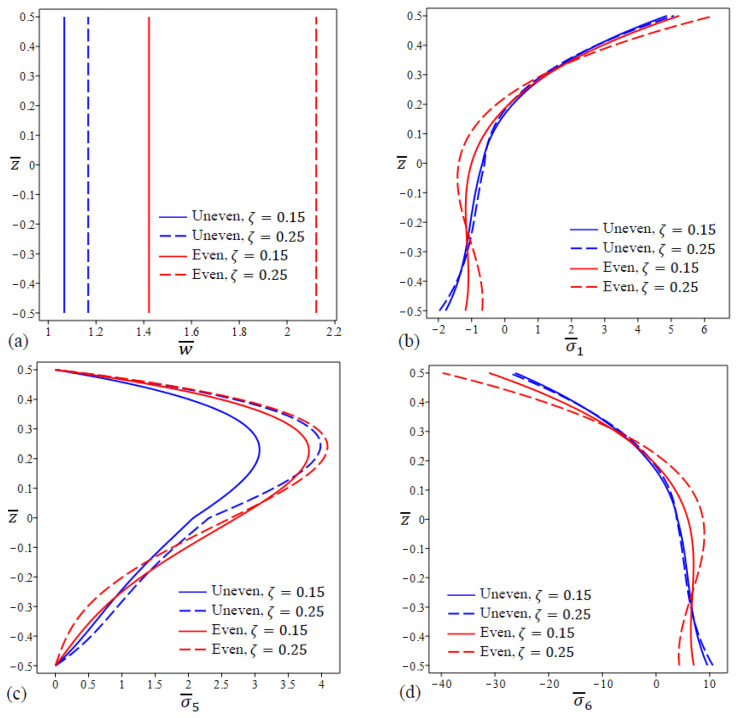
The variation in dimensionless center deflection and stresses in a square FG porous (uneven\even distribution) nanoplate for different values of ζ. (**a**) $\bar{w}$, (**b**) ${\bar{\sigma }}_{1}\;$, (**c**) ${\bar{\sigma }}_{5}$, (**d**) ${\bar{\sigma }}_{6}$.

Figure 6 shows the variation in center deflection and stresses across the thickness direction in a square FG nanoplate with uneven and even porosity distribution and two values of ζ. The smallest deflections happened for the uneven case (ζ=0.15) as demonstrated in Figure 6a. From Figure 6b, we observe that the even porosity case (ζ=0.25) causes the smallest values of normal stress ${\bar{\sigma }}_{1}$ at the plate’s lower surface and the highest values at the plate’s upper surface. The maximum value of shear stress ${\bar{\sigma }}_{5}$ at z≅0.23 are caused by the even porosity case with ζ=0.25 as shown in Figure 6c. It can be observed from Figure 6d that the in-plane stresses ${\bar{\sigma }}_{6}$ are not dependent on the porosity coefficient ζ and the porosity type at z≅−0.28 and z≅0.28.

**Figure 7:**
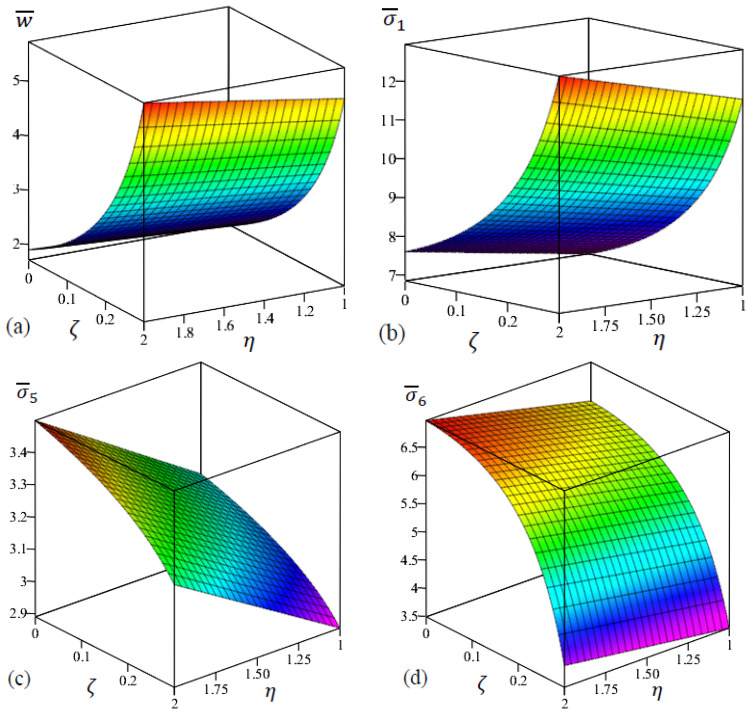
The two-dimensional distributions of the dimensionless center deflection and stresses of FG rectangular nanoplate with even porosities in terms of ζ and η. (**a**) $\bar{w}$, (**b**) ${\bar{\sigma }}_{1}\;$, (**c**) ${\bar{\sigma }}_{5}$, (**d**) ${\bar{\sigma }}_{6}$.

The two-dimensional (2D) distribution of the deflection and stresses of FG rectangular nanoplate (a/b=1/2) with even porosities in terms of ζ and η are examined in Figure 7. It can be observed from Figure 7a,b that the increasing in the porosity coefficient and the nonlocal parameter led to an increment of central deflection and normal stress ${\bar{\sigma }}_{1}$. It can be found from Figure 7c that shear stress ${\bar{\sigma }}_{5}$ is increased with the decrease in porosity coefficient ζ and the increase in the nonlocal parameter η. Furthermore, we can observe that the influence of ζ on the in-plane stress ${\bar{\sigma }}_{6}$ is more significant with the increment of η as shown in Figure 7d.

**Figure 8:**
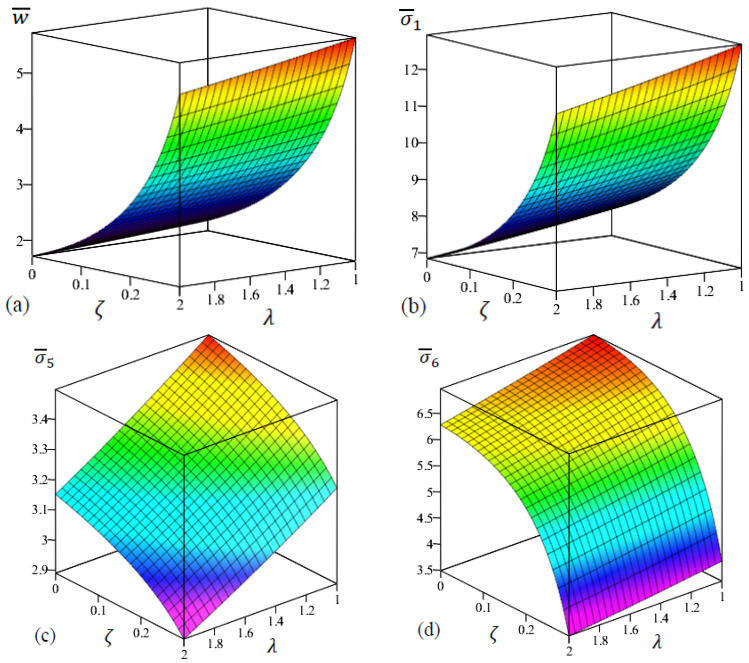
The two-dimensional distributions of the dimensionless center deflection and stresses of FG rectangular nanoplate with even porosities in terms of ζ and λ. (**a**) $\bar{w}$, (**b**) ${\bar{\sigma }}_{1}\;$, (**c**) ${\bar{\sigma }}_{5}$, (**d**) ${\bar{\sigma }}_{6}$.

Figure 8 exhibits the 2D distribution of the center deflection and stresses of FG rectangular nanoplate (a/b=1/2) with even porosities in terms of ζ and λ. The increase in the porosity coefficient with the decrease in the length scale parameter led to an increment of central deflection and normal stress ${\bar{\sigma }}_{1}$ as shown in Figure 8a,b. From Figure 8c,d, we can see that shear stress ${\bar{\sigma }}_{5}$ and in-plane stress ${\bar{\sigma }}_{6}$ are increased with the decrease in porosity coefficient and length scale parameter.

**Figure 9:**
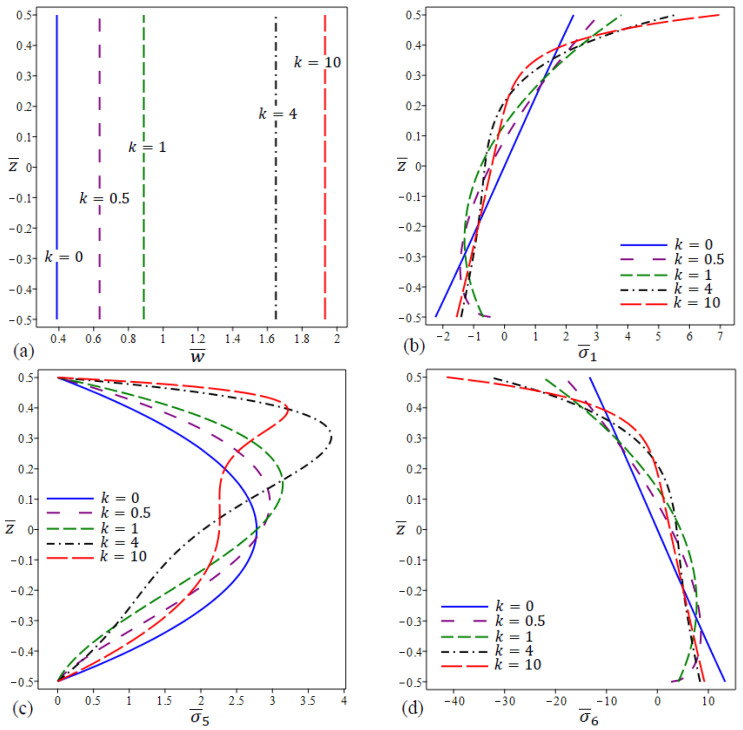
The variation in dimensionless center deflection and stresses in a square FG porous (even distribution) nanoplate for different values of k. (**a**) $\bar{w}$, (**b**) ${\bar{\sigma }}_{1}\;$, (**c**) ${\bar{\sigma }}_{5}$, (**d**) ${\bar{\sigma }}_{6}$.

In Figure 9, the variation in center deflection and stresses across the thickness direction in a square FG nanoplate with even porosity distribution and different values of the power law exponent is plotted. Figure 9a shows that the deflection increases as k increases for fixed ζ=0.15, λ=1 and η=2. The in-plane normal stresses ${\bar{\sigma }}_{1}$ of the FG porous nanoplate are tensile at the upper surface and compressive at the lower surface, as depicted in Figure 9b. The homogeneous FG porous nanoplate yields the highest compressive stresses at the lower surface and the lowest tensile stresses at the upper surface. Figure 9c captures the non-dimensional shear stress along the plate thickness of various FG porous nanoplates for different power law exponent k values. For the homogeneous FG porous nanoplate, the maximum results occur at a point on the mid-plane. The maximum values for the other FG porous nanoplate, on the other hand, are at different locations of the FG porous nanoplate. For k=4, the highest magnitude of the FG porous nanoplate is obtained.

Finally, Figure 9d illustrates the through thickness distribution of in-plane tangential stresses ${\bar{\sigma }}_{6}$ of FG porous nanoplate for various exponential factors. The in-plane tangential stresses, unlike in-plane normal stresses$\;{\bar{\sigma }}_{1\;}$, are tensile at the lower surface and compressive at the upper surface. The highest compressive stress is found at the upper surface of the FG porous nanoplate for k=10.

## 6. Conclusions

This paper is interested in developing the nonlocal strain gradient theory for the analysis of the bending of FG porous nanoplates. A four-variable shear deformation theory is used for the modeling of the FG porous nanoplate. Two different porosity distributions are considered in this paper. The equilibrium equations are described in detail and derived utilizing the virtual work concept and Navier’s procedure. The nonlocal and strain gradient parameters, porosity factor, length-to-thickness ratio, and aspect ratio are all explored. Comparison studies are provided. Additional results are provided to serve comparison purposes. The major findings are as follows:

- The presence of a porosity factor has a significant impact on the static response of the FG nanoplate and FG nanoplates with unevenly distributed porosities deflect less than those with evenly distributed porosities;
- The impact of the nonlocal parameter on the central deflection and stresses is opposite to that of the response of strain gradient parameter under the same conditions;
- The impact of aspect ratio a/b on the central deflection is much larger than that of the side-to-thickness ratio a/h;
- The results in the tables and figures indicate that the decreasing in the length scale parameter λ increases the center deflection and stresses;
- The center deflection and stresses increase by the increase in the nonlocal parameter η for all types of FG porous nanoplates. It implies that the nonlocal parameter may be capable of reducing the stiffness of FG porous nanoplates.

## Figures and Tables

**Table 1:** Dimensionless deflection and stresses in a square FG nanoplate (a/h=10).

k	η	Method	$\bar{w}$	${\bar{\sigma }}_{1}$	${\bar{\sigma }}_{5}$	${\bar{\sigma }}_{6}$
0	0	Sobhy [58]	2.9603	19.9550	2.4618	10.7450
		Hoa et al. [59]	2.9607	19.9433	2.3873	10.7387
		Present	2.9606	19.9432	2.3857	10.7387
	2	Sobhy [58]	5.2977	35.7108	4.4056	19.2289
		Hoa et al. [59]	5.2983	35.6900	4.2723	19.2177
		Present	5.2981	35.6898	4.2694	19.2176
0.5	0	Sobhy [58]	5.4971	29.6544	2.4559	4.4493
		Hoa et al. [59]	5.4816	29.3487	2.2354	4.4899
		Present	5.4974	29.6351	2.3794	4.4474
	2	Sobhy [58]	9.8374	53.0686	4.3950	7.9624
		Hoa et al. [59]	9.8096	52.5215	4.0003	8.0350
		Present	9.8380	53.0340	4.2580	7.9589

**Table 2:** Dimensionless deflection $\bar{w}$ in a square FG nanoplate (a/h=10).

a/h	η	Method	k				
			**0**	**0.5**	**1**	**4**	**10**
4	0	Hoa et al. [59]	3.7905	5.6097	7.1689	11.0892	13.5096
		Present	3.7864	5.6546	7.2842	11.5987	13.9086
	0.5	Hoa et al. [59]	3.9775	5.8866	7.5227	11.6364	14.1762
		Present	3.9732	5.9336	7.6437	12.1711	14.5949
	1	Hoa et al. [59]	4.5387	6.7171	8.5840	13.2781	16.1762
		Present	4.5338	6.7708	8.7221	13.8881	16.6540
	1.5	Hoa et al. [59]	5.4740	8.1012	10.3528	16.0142	19.5096
		Present	5.4680	8.1660	10.5194	16.7500	20.0858
10	0	Hoa et al. [59]	2.9607	4.5292	5.8701	8.7307	10.0194
		Present	2.9606	4.5371	5.8895	8.8148	10.0870
	0.5	Hoa et al. [59]	3.1068	4.7527	6.1598	9.1615	10.5139
		Present	3.1067	4.7610	6.1802	9.2498	10.5848
	1	Hoa et al. [59]	3.5451	5.4233	7.0289	10.4540	11.9972
		Present	3.5450	5.4327	7.0521	10.5547	12.0781
	1.5	Hoa et al. [59]	4.2756	6.5408	8.4773	12.6082	14.4694
		Present	4.2755	6.5522	8.5053	12.7297	14.5670
100	0	Hoa et al. [59]	2.8042	4.3255	5.6252	8.2859	9.3613
		Present	2.8042	4.3255	5.6254	8.2868	9.3620
	0.5	Hoa et al. [59]	2.9426	4.5389	5.9028	8.6948	9.8232
		Present	2.9426	4.5390	5.9030	8.6957	9.8239
	1	Hoa et al. [59]	3.3577	5.1793	6.7356	9.9215	11.2091
		Present	3.3577	5.1794	6.7358	9.9225	11.2099
	1.5	Hoa et al. [59]	4.0496	6.2466	8.1236	11.9660	13.5189
		Present	4.0496	6.2467	8.123939	11.9671	13.5199

**Table 3:** Dimensionless deflection $\bar{w}$ in a square FG porous nanoplate (k=2, a/h=10).

η	λ	Perfect	Even		Uneven	
		ζ=0	ζ=0.15	ζ=0.25	ζ=0.15	ζ=0.25
0	0	0.7573	1.1081	1.6539	0.8400	0.9098
	1	0.6325	0.9254	1.3812	0.7015	0.7599
	2	0.5429	0.7944	1.1858	0.6023	0.6523
	4	0.4232	0.6192	0.9242	0.4694	0.5084
1	0	0.9068	1.3268	1.9804	1.0058	1.0894
	1	0.7573	1.1081	1.6539	0.8400	0.9098
	2	0.6501	0.9512	1.4198	0.7211	0.7811
	4	0.5067	0.7414	1.1066	0.5621	0.6088
2	0	1.0563	1.5455	2.3068	1.1717	1.2690
	1	0.8821	1.2907	1.9265	0.9785	1.0598
	2	0.7572	1.1081	1.6539	0.8400	0.9098
	4	0.5902	0.8636	1.2890	0.6547	0.7091
4	0	1.3552	1.9830	2.9598	1.5033	1.6282
	1	1.1318	1.6561	2.4718	1.2555	1.3598
	2	0.9716	1.4217	2.1220	1.0778	1.1674
	4	0.7573	1.1081	1.6539	0.8400	0.9098

**Table 4:** Dimensionless axial stress ${\bar{\sigma }}_{1}$ in a square FG porous nanoplate (k=2, a/h=10).

η	λ	Perfect	Even		Uneven	
		ζ=0	ζ=0.15	ζ=0.25	ζ=0.15	ζ=0.25
0	0	3.6067	4.0854	4.8992	3.8081	3.9583
	1	3.0121	3.4119	4.0915	3.1803	3.3058
	2	2.5858	2.9290	3.5125	2.7302	2.8379
	4	2.0154	2.2829	2.7376	2.1279	2.2119
1	0	4.3186	4.8918	5.8662	4.5597	4.7396
	1	3.6067	4.0854	4.8992	3.8081	3.9583
	2	3.0963	3.5072	4.2058	3.2691	3.3981
	4	2.4132	2.7335	3.2780	2.5480	2.6485
2	0	5.0306	5.6982	6.8333	5.3114	5.5210
	1	4.2013	4.7588	5.7068	4.4358	4.6108
	2	3.6067	4.0854	4.8992	3.8081	3.9583
	4	2.8110	3.1841	3.8184	2.9680	3.0851
4	0	6.4544	7.3110	8.7674	6.8148	7.0836
	1	5.3904	6.1058	7.3221	5.6913	5.9159
	2	4.6275	5.2417	6.2858	4.8859	5.0787
	4	3.6067	4.0854	4.8992	3.8081	3.9583

**Table 5:** Dimensionless shear stress ${\bar{\sigma }}_{5}$ in a square FG porous nanoplate (k=2, a/h=10).

η	λ	Perfect	Even		Uneven	
		ζ=0	ζ=0.15	ζ=0.25	ζ=0.15	ζ=0.25
0	0	2.1857	2.1271	2.0595	1.9621	1.8000
	1	1.8254	1.7765	1.7200	1.6386	1.5033
	2	1.5670	1.5251	1.4766	1.4067	1.2905
	4	1.2213	1.1886	1.1508	1.0964	1.0058
1	0	2.6171	2.5470	2.4660	2.3494	2.1553
	1	2.1857	2.1271	2.0595	1.9621	1.8000
	2	1.8763	1.8261	1.7680	1.6844	1.5453
	4	1.4624	1.4233	1.3780	1.3128	1.2044
2	0	3.0485	2.9669	2.8726	2.7367	2.5106
	1	2.5460	2.4778	2.3990	2.2855	2.0967
	2	2.1857	2.1271	2.0595	1.9621	1.8000
	4	1.7035	1.6579	1.6052	1.5292	1.4029
4	0	3.9114	3.8067	3.6856	3.5113	3.2212
	1	3.2666	3.1791	3.0780	2.9324	2.6902
	2	2.8043	2.7292	2.6424	2.5174	2.3094
	4	2.1857	2.1271	2.0595	1.9621	1.8000

**Table 6:** Dimensionless shear stress ${\bar{\sigma }}_{6}$ in a square FG porous nanoplate (k=2, a/h=10).

η	λ	Perfect	Even		Uneven	
		ζ=0	ζ=0.15	ζ=0.25	ζ=0.15	ζ=0.25
0	0	5.44216	5.10538	4.1978	5.4135	5.3822
	1	4.5450	4.2637	3.5058	4.5211	4.4949
	2	3.9018	3.6603	3.0096	3.8812	3.8588
	4	3.0410	2.8529	2.3457	3.0250	3.0075
1	0	6.5164	6.1131	5.0264	6.4821	6.4446
	1	5.4421	5.1054	4.1978	5.4135	5.3822
	2	4.6720	4.3829	3.6037	4.6474	4.6205
	4	3.6413	3.4160	2.8087	3.6222	3.6018
2	0	7.5906	7.1209	5.8550	7.5507	7.5070
	1	6.3393	5.9470	4.8898	6.3059	6.2694
	2	5.4422	5.1054	4.1978	5.4135	5.3822
	4	4.2416	3.9791	3.2717	4.2193	4.1948
4	0	9.7391	9.1364	7.5122	9.6878	9.6317
	1	8.1336	7.6303	6.2738	8.0908	8.0439
	2	6.9825	6.5504	5.3859	6.9458	6.9055
	4	5.4422	5.1054	4.1978	5.4135	5.3822

## Data Availability

Not applicable.

## Appendix A

$$a_{11}=A_{1}\alpha^{2}+A_{24}\beta^{2},a_{12}=(A_{4}+A_{24})\alpha \beta ,a_{13}=-\alpha [A_{2}\alpha^{2}+\left(A_{5}+2A_{25}\right)\beta^{2}],\;a_{14}=-\alpha [A_{3}\alpha^{2}+\left(A_{6}+2A_{26}\right)\beta^{2}],a_{15}=-\alpha A_{7},a_{22}=A_{24}\alpha^{2}+A_{8}\beta^{2},\;a_{23}=-\beta [A_{9}\beta^{2}+\left(A_{5}+2A_{25}\right)\alpha^{2}],a_{24}=-\beta [A_{10}\beta^{2}+\left(A_{6}+2A_{26}\right)\alpha^{2}],\;a_{25}=-\beta A_{11},a_{33}=A_{12}\alpha^{4}+2\left(A_{14}+2A_{27}\right)\alpha^{2}\beta^{2}+A_{17}\beta^{4},\;a_{34}=A_{13}\alpha^{4}+2\left(A_{15}+2A_{28}\right)\alpha^{2}\beta^{2}+A_{18}\beta^{4},a_{35}=A_{16}\alpha^{2}+A_{19}\beta^{2},\;a_{44}=A_{20}\alpha^{4}+A_{32}\alpha^{2}+2\left(A_{21}+2A_{29}\right)\alpha^{2}\beta^{2}+A_{30}\beta^{4}+A_{22}\beta^{4},\;a_{45}=(A_{22}+A_{33})\alpha^{2}+(A_{23}+A_{31})\beta^{2},a_{55}=-(A_{34}\alpha^{2}+A_{35}\beta^{2}+A_{36}).$$
